# Exploring Barriers To Sexual and Reproductive Healthcare for Latina Women in South Carolina

**DOI:** 10.1007/s10903-025-01736-4

**Published:** 2025-07-12

**Authors:** Isabelle Theodossiou, Kristen McLean, Beth Sundstrom, Cara Delay

**Affiliations:** 1 College of Charleston, Charleston, USA; 2 University of South Carolina, Columbia, USA

## Abstract

Migrant and minority women in the United States face a high likelihood of experiencing poor reproductive health outcomes. Hispanic/Latina women are an especially high-risk population. Comprehensive and high-quality sexual and reproductive healthcare (SRHC) is desperately needed among this population, yet many Latina women face substantial barriers in accessing care. This study builds upon the three-delays model to better understand why Latina women experience delays in accessing quality SRHC in the state of South Carolina. Data for this study were drawn from semi-structed interviews with 14 adult women identifying as either Hispanic or Latina. Findings reveal that structural factors, including long distances to facilities, high costs of care, and difficulties navigating local healthcare and insurance systems, served as barriers to accessing quality SRHC. Socio-cultural factors, such as communication challenges and low cultural competency among healthcare providers, further inhibited access to care, even among women who spoke fluent English. These findings indicate a need for SRHC education and services to be more culturally-centered, by accounting for cultural knowledge and historical dynamics, and by giving patients more agency with respect to their care. Regarding the three-delays model, future applications should seek to better incorporate preventive services and consider that perceptions of quality SRHC are both individually- and contextually-mediated. This will be an important step toward developing policies and programs that are appropriately tailored to specific populations’ cultural backgrounds and contextual needs.

## Background

Hispanic/Latina women in the United States are more likely than their non-Hispanic white counterparts to experience poor reproductive outcomes, including preterm birth, low birthweight, and delayed prenatal care [1]. Racial-ethnic disparities have also been documented for teenage births, contraceptive use, sexually-transmitted infections (STIs), and reproductive cancers [2]– [3]. Access to comprehensive and high-quality sexual and reproductive healthcare (SRHC) is desperately needed to reduce these disparities [4], yet many Latina women face substantive barriers in obtaining care [3].

For many Latina women, the risk of poor sexual and reproductive health outcomes is exacerbated by their migrant status [5]– [6]. Key obstacles to accessing quality SRHC for migrant and minority women include cost, language and cultural barriers, low health literacy, mistrust of the medical system, and bureaucratic challenges [6–12]. Smith and colleagues [13] note how policies such as the Hyde Amendment Codification Act, which prohibits funding for abortion through federal insurers like Medicaid, negatively impact low-income women and women of color, who are more likely to use federally funded insurance. Additional insight into this topic is particularly timely in light of the overturning of *Roe v. Wade* in 2022, which experts predict will exacerbate inequalities in access to SRHC and reproductive health outcomes [14]– [15], especially for Latina women given their heavy concentration in states restricting or banning abortion [16]. The Covid-19 pandemic has posed additional challenges in addressing reproductive health disparities, with its disproportionate impact on immigrant and Latinx communities in the U.S [4, 17].

Further research is needed to better understand barriers to SRHC in the southeastern United States in particular, given the region’s increasingly high density of Latinx populations [13, 18]– [19]. From 1990 to 2005, the Latinx population in Arkansas, Alabama, Georgia, North Carolina, South Carolina, and Tennessee grew by around 447%, with South Carolina witnessing the largest growth [20]. In 1990, there were 30,551 Latinxs in the state; by 2005, that figure had grown to 135,041 [20], and by 2024 it reached 410,912 (7.5% of the population) [21]. Despite these trends, little is known about access to SRHC among foreign-born and second-generation Latinas in this part of the country [18]. South Carolina ranks among the top states in the country in terms of maternal vulnerability ratings [22]; in 2024 the state earned a grade of “F” by a March of Dimes report, for its preterm birth rate of 11.6% [23]. The maternal mortality rate is 32.2 per 100,000 births, and an estimated 16.1% of the population receives inadequate prenatal care [23]. As of 2013, the fertility rates of Latinas in South Carolina were more than double those of non-Latina white and Black women in the state, but Latina women were significantly less likely to receive prenatal care compared to white and Black women [24]. A 2023 March of Dimes study demonstrated that almost 28% of Latina women in South Carolina received inadequate prenatal care [25]. This study seeks to highlight prominent challenges faced by Latinas in accessing SRHC in South Carolina, where women of color experience disparities in sexual and reproductive health outcomes compared to women nationally [26–28].

### Theoretical/Conceptual Framework

This study draws upon the three-delays model, which was originally developed to account for maternal mortality in Africa [29]. The model suggests that women face three types of “delays” in accessing obstetric care: (1) delays in deciding to seek care, (2) in reaching a medical facility, and (3) in receiving care. Delays related to factors involved in the decision-making process include whether an individual has sufficient information to recognize when an emergency is imminent or whether they perceive the cost and distance to a health facility as prohibitive. Factors that impede one’s ability to reach care tend to include structural barriers, such as lack of transportation or poor road infrastructure, whereas barriers to receiving quality services upon arrival at a hospital or clinic often stem from poorly equipped facilities, unskilled clinicians, and/or discrimination by facility staff.

The three-delays model has since been adapted to explain barriers to SRHC for migrant populations in high-income settings. Binder and colleagues [30] introduce the “maternal migrant effect” to suggest that migrant women tend to be influenced by maternal health experiences from their country of origin. This body of work reveals that in high-income contexts socioeconomic and infrastructure barriers pose less of an issue whereas socio-cultural factors, including communication challenges and distrust of medical personnel, heavily inform women’s SRHC-seeking behaviors [30–32]. A study examining barriers to healthcare access among Latinx migrants in Michigan used the model to highlight the importance of attending to political climate, particularly as related to anti-immigrant sentiment [5]. We employ and build upon this model to explore the reproductive healthcare challenges facing Latina women in South Carolina by distinguishing between emergency and preventive services and by demonstrating that perceptions of “quality” SRHC are individually- and contextually-mediated.

## Methods

This study is part of a larger research project conducted between 2016 and 2019 to examine the reproductive healthcare experiences of 70 South Carolina women [13]. Our analysis offers insights from a sub-sample of women who self-identified as Hispanic and/or Latina. The study protocol received approval from the College of Charleston Institutional Review Board and all participants went through a process of informed consent before agreeing to take part in the study.

### Participants

Women aged 18 years or older and who lived in the counties of Charleston, Beaufort, Dorchester, Colleton, and Richland were eligible to participate. To recruit women, flyers were placed in community-central locations such as churches, libraries, and clinics (primary-care physician and OB/GYN offices) and were disseminated via Facebook. Women were also recruited at community events such as markets or fairs. Purposive convenience sampling and snowball sampling (where participants were asked for referrals) were used to recruit a sample that was diverse and representative of the South Carolina population.

The findings detailed below concern a sub-sample of 14 Hispanic/Latina-identifying women between the ages of 19 and 53. Of these women, eight were foreign-born, having migrated from various countries such as Mexico, Honduras, El Salvador, Guatemala, and the Dominican Republic. Ten women had completed at least some college and were either currently employed (*n* = 4) or students (*n* = 9). Five were married.

### Data Collection

Semi-structured face-to-face interviews were conducted in English or Spanish, depending on participants’ preference, by researchers trained in qualitative methodology. This approach facilitated flexibility; interviewers prepared a series of questions but also changed the order of questions and added or deleted questions as needed [33].

Interviews lasted approximately one to two hours and were completed either in person at a private location or over the phone. Questions focused on participants’ experiences with SRHC in South Carolina. Sample questions included: Can you describe any challenges you’ve had accessing reproductive healthcare? How have you communicated/expressed yourself with reproductive healthcare professionals? What was it like being pregnant and having a child in your community? All interviews were audio-recorded, with consent, and then translated (if necessary) into English and transcribed.

### Analysis

The first two authors independently reviewed and explored themes from interview transcripts using deductive and inductive approaches. They identified themes stemming from Binder and colleague’s [30] “migration three delay model” (e.g., broken trust due to past negative SRHC experiences, mutual language discordance, etc.) and also employed thematic analysis, in which themes were derived directly from the data [34]. The authors developed a list of codes for organizing the data into conceptual categories and themes, and once consensus was reached regarding codes and their definitions, applied a finalized codebook to all transcripts. In our analysis we introduced an intersectional lens by considering factors beyond ethnicity, such as participant age and country of origin.

## Results

Data analysis revealed a number of factors influencing Latina women’s decision-making and ability to access quality SRHC. These factors fall into three main categories: (1) recognition of a need for care, (2) structural impediments to care-seeking, and (3) issues regarding the importance of culture-centered care.

### Recognizing a Need for SRHC

One central theme was the perceived need for SRHC; the majority of women reported that they would not seek care from a practitioner unless they had a specific medical reason. A disregard for preventive services was especially prominent among older, foreign-born participants, none of whom had sought care from a gynecologist prior to becoming pregnant. As a 53-year-old woman from El Salvador explained, regular doctor’s visits were unnecessary because she lived a healthy lifestyle. While younger participants displayed more mixed attitudes toward preventive care, more than half of these women reported going without regular check-ups. As a 21-year-old college student from New York explained, “We don’t have a lot of health issues, so we just don’t regularly go to the doctor.”

Our findings also revealed a general lack of education about sexual and reproductive health issues, which may have impacted healthcare seeking. A majority of women noted an absence of conversations about such topics within the home. A 39-year-old woman from Honduras, explaining a lack of discussion surrounding pregnancy in her family said, “My mom was the one who was pregnant, and suddenly she would go out and when she came back, she came back with the baby.” Women also noted how topics like contraception and menstruation were considered culturally taboo, with one woman from Mexico stating that she had only begun to discuss contraceptives with her mother as an adult, once she relocated to the United States. Younger participants reported having access to more resources to learn about reproductive health issues; for instance, they often learned about these topics at school, in the media, or from peers. Several women noted that while in their countries of heritage it was culturally normative to have many children and to begin childbearing at a young age, they were influenced by American childbearing norms (fewer children at a later maternal age), which impacted their decisions to seek family planning services.

As our interviews revealed, conservative religious beliefs further influenced the healthcare-seeking behaviors of participants. As one woman explained, “I feel like a lot of Latinos are very Catholic so that topic [sex] is never brought up unless it’s in a shameful way, like, ‘don’t do this.’” Three of the women in the study said they chose not to make use of contraception despite being educated about their options for religious reasons. Several others reported having utilized contraception at some point in their lives but feeling conflicted about the decision to do so. As a woman from Mexico stated, “Yes they [contraceptives] seem good to me, but there’s also, when you’re in a certain religion…you might sometimes start to feel bad, because they say, my religion doesn’t allow this… There’s a feeling of guilt.”

### Structural Impediments to Accessing SRHC

Participants reported experiencing substantial structural barriers in accessing SRHC. A few women noted long distances to health facilities, which made accessing care financially burdensome or simply not feasible. This often went hand-in-hand with issues related to insurance and cost of care, as women had to travel further to facilities that accepted their insurance. A 40-year-old woman from Mexico recounted a delay in seeking care for an obstetric emergency during her pregnancy because she could not decide between traveling an hour away to the hospital that accepted her insurance or presenting to the closest facility. Eventually, facing a hemorrhage, she resorted to calling an ambulance, which took her to a nearby clinic. As she recounted, “There wasn’t time for anything. In fact, the doctor didn’t have permission to receive me. But…she attended to me there because there wasn’t time.” In this scenario, having to weigh financial cost against timeliness of care led to further delay, which wasted valuable time and made a stressful obstetric complication more dangerous.

Some women also described challenges navigating local healthcare systems following relocation, either from a foreign country to the U.S., or within the U.S. A few of the women, for instance, reported experiencing disrupted care after relocating to South Carolina to attend college. A 19-year-old from Florida explained the reason behind her delay in seeking SRHC, saying, “I think it was my parents didn’t really know a lot about the area. It also could have been getting everything transferred, like all of our health insurance information.” A 21-year-old from Colombia explained, “Definitely when we first came here [the United States] the language barrier was huge but also getting to understand the system.” Several of the younger women specifically referenced difficulties in accessing care upon “aging out” of their parent’s health insurance. A 19-year-old explained that she had positive experiences with reproductive healthcare until she turned 18, and “then I got it [insurance] taken away and now I can only go to the gynecologist once a year. And I have to pay for my own medication.”

Navigating the American health insurance system also created confusion and stress that sometimes contributed to delayed care-seeking according to study participants. One woman explained that it wasn’t always clear if her insurance provider would cover certain services. Another reported being billed incorrectly by her insurance provider and having to spend valuable time on the phone clarifying mistakes, an experience that is common in the U.S. but may present a heightened obstacle for those who already face language barriers and/or are unfamiliar with the U.S. healthcare system.

### The Importance of Culture-Centered SRHC

Upon reaching health facilities, some participants reported facing communication and/or discrimination-related challenges that negatively impacted their perceived quality of care. These barriers were experienced most severely by women who spoke Spanish as their primary language; these women reported feeling that their healthcare provider did not adequately listen or attend to them during appointments. In one case, a participant from El Salvador described a provider failing to adequately acknowledge her pain, explaining, “I told the doctor that I felt bad, but the doctor didn’t listen to me. And so she told me, ‘take Tylenol…for the pains you’re having’ and I said that I [still] didn’t feel well.”

Participants interpreted communication-related barriers in various ways. A 46-year-old woman from Mexico blamed poor quality healthcare encounters on her limited English language skills, saying, “At that time I did not know any English, and I didn’t know what I could say, because they weren’t going to understand me.” It is unclear why a translator was not present for her appointments, but even for women who used a translator, communication barriers sometimes persisted. One woman described a provider who spoke directly to her in English rather than utilizing the translator, who had to interject to remind the provider that the patient did not understand what was being said.

While language barriers presented a challenge for some women, even participants who spoke fluent English at times felt that healthcare providers did not sufficiently listen to or understand them. Several of these women recounted instances when they felt as though their healthcare provider “didn’t really care” or were attempting “to get [them] in and out.” A 19-year-old from Florida felt that her provider dismissed her concerns when she presented with a lump in her breast, despite the fact that—and as she had communicated to her doctor— breast cancer runs in her family. This woman attributed her experience to discrimination against Hispanic/Latinx populations when they seek care at clinics run by predominantly white providers, who she felt labeled them as “people who don’t really know anything.”

According to several participants, communication problems also stemmed from cultural differences between healthcare providers and Hispanic/Latina-identifying patients. As a college student from New York explained, “The cultural thing, I feel like it does play a role… Latino cultures, it’s very different from a white culture.” She believes that most SRHC services cater to a primarily white American clientele, which means that services may not be “as useful” to those of Hispanic background. She conveyed her frustrations, saying, “I wish there was some sort of training or something to help with that. I guess just being more understanding of the different cultural differences that may exist between a doctor and their patient.” Cultural differences between healthcare staff and patients may have factored into women’s beliefs that they experienced a “distant relationship” with their healthcare provider. In general, the younger participants—those primarily raised in the U.S.—seemed to expect a more personal relationship with their providers compared to older women, and as a result, tended to be less satisfied with their quality of care.

## Discussion

This study examined the major barriers Hispanic/Latina women face when accessing SRHC in South Carolina, guided by an adapted version of the three delays model. In line with other studies conducted with immigrant and minority populations in high-income countries, our findings indicate that numerous structural factors prevented participants from accessing quality SRHC, among them long distances to facilities, high costs of care, and difficulties navigating local healthcare and insurance systems [5, 7, 35–38]. Though structural impediments to accessing SRHC may be reduced in higher-resource settings as the literature suggests [30], our findings indicate that they can be substantial nonetheless and are often tied to particular social and bureaucratic environments. When deciding which healthcare facilities to utilize, women had to consider both geographical factors and whether the facility would accept their insurance. We also highlight particular instances when young women may be especially vulnerable to systems-related challenges, such as when they “age out” of a parent’s insurance plan or after they relocate to attend college in another state.

Navigating healthcare in the U.S. can be especially confusing compared to other contexts because different states have different policies, care is often fragmentary, and the system is ridden with hidden costs [39]. It is thus important that targeted information and services are provided to support women in navigating these challenges. Morales- Alemán and colleagues [18] suggest that community-level outreach interventions such as community health worker models [40] could prove vital for Latinx communities in the U.S. South. To assist with cost and distance-related barriers, researchers recommend promoting an increased presence of healthcare services in underserved communities, by providing incentives to attract healthcare workers to these areas or developing telehealth programs [5, 41].

Similar to other literature on the SRHC experiences of immigrant women in high-income settings, our findings indicate that socio-cultural factors, including communication challenges and low cultural competency among healthcare providers, negatively influenced care encounters [5, 30, 42–44]. Women described experiencing discrimination, a lack of deep listening on the part of providers, and disappointment that relationships with providers were not more personal. For some, these challenges were related to limited language proficiency and poor-quality translation services. However, even more culturally-fluent women born in the U.S. were dissatisfied with clinician encounters, as seen in other studies of young Latinas [18].

Researchers indicate the need for SRHC to be “culture-centered” [45]– [46] by accounting for cultural knowledge, historical dynamics, and by positioning beneficiaries with agency, for instance, by relying upon participatory approaches [47]– [48]. This can be done by promoting interventions and policies that seek to hire service providers from local communities or those with more diverse language and socio-cultural backgrounds [48]– [49]. To address communication-related challenges, researchers encourage training clinicians to better interact with interpreters [42, 44] and employing translators who are properly trained within the medical field to avoid translation errors and violations of confidentiality [44, 50]– [51]. Another approach suggests the provision of cultural humility training for healthcare workers as a way to foster more appropriate and compassionate care [18, 44, 52]. Initiatives to train healthcare staff on the specific needs of migrant populations have proved fruitful [53].

Our findings also compel us to suggest considerations for future applications of the three delays model. The original model [29] was used to identify factors contributing to delays in accessing emergency obstetric care, and as such, has primarily been applied in the context of healthcare treatment rather than prevention. We suggest that future iterations of the model seek to incorporate preventive SRHC, as our findings indicate that the perceived need for such services may be lower among certain groups. Women in the study, for instance, indicated that they did not need preventive services, such as annual gynecological check-ups, as they associated such services with women who lead unhealthy lifestyles. They also indicated a lack of comprehensive sexual and reproductive health education—e.g., given that topics such as sex and contraception are considered culturally taboo—a finding that aligns with other research conducted among Hispanic/Latina women [42, 49, 54].

Scholars have argued that low uptake of SRHC, and particularly preventive services, may stem in part from the “maternal migration effect” [30], which describes a conflict between pre-migration and post-migration maternity care experiences and expectations. Seeking preventive services may be less common in one’s country of heritage and thus not sought after migration. However, research with Latina migrant populations finds that women do in fact value and prioritize prevention when it comes to their sexual health [42]. Thus, a lack of SRHC-seeking may have more to do with expectations surrounding care. One study of Mexican migrant women in New York found that negative past encounters with healthcare providers, compounded by a lack of SRHC knowledge, led to lower rates of SRHC utilization [7]. As our findings demonstrate, what women consider “quality” care varies based upon one’s age, life experiences, and family dynamics. Indeed in some instances, seeking healthcare may not be in a woman’s best interest, contrasting the primarily Western notion that all care is necessarily “good” care. Practitioners and policy makers must recognize that women are able to negotiate the meaning and value of healthcare for themselves and have a right to exercise reproductive autonomy [13].

### Limitations

Our findings should be interpreted in light of several limitations. First, this study includes only 14 interviews because it involves a sub-sample (Latina-identifying women) from a larger research project examining reproductive healthcare experiences in South Carolina. Due to our small sample size and use of a convenience sampling approach, findings cannot be generalized to all Latina populations in the United States, which may experience diverse immigrant trends or face unique political or institutional barriers to SRHC. Second, our sample is weighted towards younger individuals (ages 19–22), representing a mix of U.S. and foreign-born women. Future work should seek to include a more representative sample of first- and second-generation Latina women to more fully understand their broad range of experiences. We also call for future research to include more intersectional analyses, considering not only race/ethnicity, migration status, and age but also socioeconomic status, gender identity, region (rural/urban), and sexual orientation.

### New Contribution to the Literature

As one of the first studies focusing on Latina women and SRHC in South Carolina [24, 55–59], this research contributes to understanding barriers to SRHC among this population in the U.S. South, where oppressive systems of power have historically disadvantaged minority women [13]. Additionally, it expands upon the three-delays model as applied to migrant women in high-income settings. To our knowledge, this is the first study to apply this model to reproductive healthcare experiences of Latina women in the U.S. South. This is an important first step toward developing policies and interventions that are culturally-relevant and adapted to this populations’ linguistic and socio-economic needs.

## Data Availability

The data that support the findings of this study cannot be shared openly in order to protect study participant confidentiality. De-identified data, however, may be available from the authors upon reasonable request and with permission of the College of Charleston Institutional Review Board.
